# Personalized Genetic Diagnosis of Congenital Heart Defects in Newborns

**DOI:** 10.3390/jpm11060562

**Published:** 2021-06-16

**Authors:** Olga María Diz, Rocio Toro, Sergi Cesar, Olga Gomez, Georgia Sarquella-Brugada, Oscar Campuzano

**Affiliations:** 1 UGC Laboratorios, Hospital Universitario Puerta del Mar, 11009 Cadiz, Spain; olgamadime@hotmail.com; 2 Biochemistry and Molecular Genetics Department, Hospital Clinic of Barcelona, Institut d’Investigacions Biomèdiques August Pi i Sunyer (IDIBAPS), Universitat de Barcelona, 08950 Barcelona, Spain; 3 Medicine Department, School of Medicine, Cádiz University, 11519 Cadiz, Spain; rociotorogreen@gmail.com; 4 Arrhythmia, Inherited Cardiac Diseases and Sudden Death Unit, Institut de Recerca Sant Joan de Déu, Hospital Sant Joan de Déu, University of Barcelona, 08007 Barcelona, Spain; sergi.cesar@gmail.com; 5 Fetal Medicine Research Center, BCNatal-Barcelona Center for Maternal-Fetal and Neonatal Medicine (Hospital Clínic and Hospital Sant Joan de Deu), Institut d’Investigacions Biomèdiques August Pi i Sunyer (IDIBAPS), Universitat de Barcelona, 08950 Barcelona, Spain; OGOMEZ@clinic.cat; 6 Centre for Biomedical Research on Rare Diseases (CIBER-ER), 28029 Madrid, Spain; 7 Medical Science Department, School of Medicine, University of Girona, 17003 Girona, Spain; 8 Centro de Investigación Biomédica en Red, Enfermedades Cardiovasculares (CIBER-CV), 28029 Madrid, Spain

**Keywords:** cardiology, pediatrics, congenital heart disease, genetics

## Abstract

Congenital heart disease is a group of pathologies characterized by structural malformations of the heart or great vessels. These alterations occur during the embryonic period and are the most frequently observed severe congenital malformations, the main cause of neonatal mortality due to malformation, and the second most frequent congenital malformations overall after malformations of the central nervous system. The severity of different types of congenital heart disease varies depending on the combination of associated anatomical defects. The causes of these malformations are usually considered multifactorial, but genetic variants play a key role. Currently, use of high-throughput genetic technologies allows identification of pathogenic aneuploidies, deletions/duplications of large segments, as well as rare single nucleotide variants. The high incidence of congenital heart disease as well as the associated complications makes it necessary to establish a diagnosis as early as possible to adopt the most appropriate measures in a personalized approach. In this review, we provide an exhaustive update of the genetic bases of the most frequent congenital heart diseases as well as other syndromes associated with congenital heart defects, and how genetic data can be translated to clinical practice in a personalized approach.

## 1. Introduction

Congenital cardiac alterations are a heterogeneous group of structural heart or great vessel malformations that arise during embryogenesis, usually between the third and tenth week of gestation. These types of defects give rise to congenital heart disease (CHD). The first report focused on CHD origin was published in 1950 [1], and multifactorial inheritance was hypothesized [2]. Now, CHD has a known multifactorial etiology combining genetic alterations and environmental factors [3,4].

Despite this complex origin, unraveling the potential cause of the disease will help to prevent new cases in future gestations. Therefore, a proper genetic diagnosis of CHD is crucial in personalized medicine. Recently, improvement of high throughput genetic techniques has allowed cost-effective genetic diagnosis; identification of the genetic alterations responsible for CHDs facilitates counseling regarding the recurrence risk in relatives and descendants, and prenatal and preimplantation genetic diagnosis may influence reproductive options [5]. The technical approach should be chosen according to the suspected genetic alteration and based on the clinical diagnosis. For example, if Down syndrome is suspected, the appropriate genetic approach is a karyotype, not a complete exome or genome. It is important to note that the diagnostic performance of the genetic analysis is not always successful, so that some cases remain without a definitive genetic diagnosis after the study is carried out. This situation usually occurs when a genetic alteration is identified but the interpretation is not conclusive, leaving an ambiguous role. Sometimes no genetic alteration is identified as a potential cause of disease after analysis of all known defects; thus, performing a wide-ranging analysis may allow the identification of possible causal but localized alterations in genes/chromosomes not definitively associated with suspected disease. Consequently, these alterations are again classified with an ambiguous role. Therefore, performing a complete genetic analysis is not always the answer to increase genetic yield. From our point of view, the best approach is to analyze known genes/chromosomes and perform an exhaustive interpretation before clinical translation. The analysis of unknown areas of the genome is essential to improve the genetic knowledge of CD, but it should be done in the field of research, not in clinical diagnosis.

Nowadays, CHDs require rapid diagnosis and treatment to improve their prognosis. This diagnosis can be prenatal, at the time of birth, or during the first years of childhood depending on clinical suspicions/diagnosis. More precise and earlier diagnosis allows adoption of immediate and appropriate therapeutic measures for each case, factors that increase the percentage of survival and quality of life. An estimated 65% of cases are diagnosed prenatally and 20% during the first week or month of life. Around a third of patients with CHD undergo at least one surgery during the first year of life; the results of these interventions are improving, which allows many patients to lead a relatively normal life and reach adulthood [6].

## 2. Epidemiology

CHDs are the most common form of congenital malformations (approximately one third of the total) as well as a leading cause of morbidity and mortality during childhood. CHD occurs in 0.5–0.8% of live births but this value increases among abortions and premature infants. Specifically, the prevalence ranges from 8–10 per 1000 live births before the first year of life and 12 per 1000 before the age of 16, with a higher prevalence in industrialized countries [7]. There is a higher incidence of these disorders in males than in females, as occurs with infant mortality. Likewise, differences in incidence between races have been reported, for example a higher male incidence is observed born to Asian mothers. In the Spanish population, the total incidence of CHD is 13 per 1000 live births, which is higher than the estimated worldwide incidence but similar to that of other countries such as Switzerland or Malta [8].

New diagnostic techniques allow the identification of alterations during the first days/weeks of life. This early diagnosis combined with advances in surgical procedures for CHD repair [9,10] have allowed the survival rate of patients with malformations of this type to reach almost 90% [11]. This higher survival has led to a significant increase in the number of adults with CHD, as well as an increase in the average life expectancy when the appropriate measures are adopted and with earlier diagnosis. Despite these advances, not all regions of the world have the means to implement these diagnostic techniques and treatments, which is why CHD is one of the leading causes of mortality in children under one year of age in underdeveloped countries. Women have a greater survival rate than men after a similar surgical intervention, as well as a lower rate of incidents and adverse effects in adults with corrected CHD [12].

## 3. Genetic Basis

Firstly, is important to state that genetic diagnosis is profoundly dependent on accurate clinical diagnosis. The genetic basis of CHD is currently divided into two groups: syndromic CHD and non-syndromic (or also isolated/sporadic) CHD [13]. Nowadays, most cases of CHD are diagnosed as non-syndromic, sporadic defects. Thus, neither a family history nor a clear Mendelian inheritance may be recognized. The genetic etiology of CHD is estimated in almost 30% of diagnosed cases, some of them associated with other syndromes.

Syndromic CHD is defined as CHD with other congenital anomalies and/or dysmorphic features. The alterations responsible for syndromic CHD can be aneuploidies, large deletions or duplications of DNA (>1000 nucleotides) called copy number variants (CNVs), or single nucleotide variants (rare monogenic pathogenic variants, rare oligogenic deleterious variants, and common variants) [14]. It is important to note that a single genetic defect can cause different CHDs and the same malformation can be caused by alterations in different genes. Both situations make it difficult to identify the specific origin of the malformation and complicate genetic counseling regarding transmission to descendants. In isolated CHD, pathogenic variants encode the transcription factors, signaling molecules, structural proteins and epigenetic modifiers that are essential for normal cardiac development [13,15].

### 3.1. Genetic Counselling

More than 40 years ago, Halloran et al. demonstrated that informing parents via a dedicated counseling process had a beneficial effect [16]. Recently, the beneficial effects of individualized genetic counseling in routine clinical practice have been confirmed [17]. Proper genetic testing is crucial to avoid misinterpretation of identified alterations in personalized genetic counseling. Therefore, the European Society of Human Genetics developed a policy on the advertising and provision of predictive genetic tests [18].

Avoiding a false positive/negative result is crucial to adopting appropriate measures in each family. Importantly, nearly 10% of sporadic CHDs are due to de novo alterations which are generally associated with increased morbidity [19,20]. In addition, the recurrence of another congenital defect de novo in a future pregnancy ranges between 3% and 5%, although this percentage can vary significantly depending on the specific type of heart disease as well as the responsible genetic alteration. There is also a greater association between CHD in monozygotic twins than in dizygotic twins, although a multiple pregnancy per se increases the risk of CHD [21,22,23]. Rates of CHD increase in pregnancies occurring through assisted reproduction, although it has not been clarified whether this increase is due to embryonic manipulation or to the infertility of those who must use this method of conception [24].

### 3.2. Genetic Testing

Conventional karyotyping was implemented in CHD more than 60 years ago as the first genetic testing approach [25]. This approach currently identifies the pathogenic genetic alteration in around 13% of congenital malformations at the cardiac level, most of them coinciding with aneuploidies (mainly trisomy 21, 13, and 18 and monosomy X) [26]. Fluorescent in situ hybridization was later used in CHD diagnosis, but array comparative genome hybridization (ArrayCGH) represents the main innovation in CHD diagnosis. Array-CGH technology, also called “molecular karyotyping”, detects CNVs, which are responsible for 3–20% of cases. CNVs are more frequent in patients who present other symptoms or phenotypic characteristics in addition to congenital cardiac malformation. Single-nucleotide variants can also be responsible for CHD. Monogenic diseases can follow autosomal dominant, autosomal recessive or X-linked dominant or recessive inheritance patterns [27]. These specific alterations can be identified using Sanger sequencing technology or by using new massive sequencing approaches (next generation sequencing). Thus, it is estimated that approximately 2% of CHD is due to single rare variants. Somatic mutations are not a common cause but, in some cases, could be related to the development of malformations [28,29]. Despite this advance, a comprehensive validation of all genes currently associated with CHD should be performed in order to confirm the real role of each gene. From our point of view, due to the complexity of these diseases, an exhaustive genetic analysis and proper interpretation of each variant should be done before clinical translation and adoption of personalized measures.

In 2017, Blue et al. published an excellent review focused on the timeline of CHD genetic discoveries and the genetic technologies used [30]. Continuous advances in genetic technology have provided a higher yield in genetic diagnosis of CHD although many CHD cases remain without a conclusive origin. A recent study performed in UK fetal medicine centers identified that the diagnostic yield of prenatally diagnosed CHD using quantitative fluorescence-PCR (QF-PCR), chromosome microarray (CMA), and exome sequencing (ES) was 15.6%, 13.7%, and 10.2%, respectively [31]. Another recent study concluded that most CHD diagnoses using prenatal ES are associated with extracardiac anomalies rather than isolated cardiac abnormalities [32]. Therefore, genetic testing has a low yield for sporadic CHD but should be used in patients with familial and syndromic CHD, despite the existence of a significant proportion of incidental findings and variants of unknown significance. Advances in bioinformatics tools will enable more comprehensive and accurate analysis of genetic alterations, allowing proper clinical translation of genetic data of ambiguous significance.

As above mentioned, genetic factors are the predominant cause of CHD whereas epigenetic (mainly DNA methylation, histone modification and post-translational mechanism due to microRNAs) and environmental factors (toxic agents, such as tobacco or alcohol, chemicals, such as organic solvents or pesticides, drugs, such as antiepileptics, folate antagonists and dihydrofolate reductase inhibitors, and viral infections) are also important contributors. Genetic mechanisms underlying CHD development are complex and remain elusive using current genetic approaches. Despite advances in mass sequencing, two main limitations exist. The first is the interpretation of genetic data; an exhaustive genetic understanding of identified alterations allows proper transition into clinical practice, facilitating diagnosis and adoption of therapeutic measures. Hence, interpretation of the variants often requires additional validation including functional studies and genotype–phenotype family segregation. In many families, genetic testing identifies a potential genetic alteration responsible for CHD, but lack of data impedes a definitive genetic diagnosis. These alterations remain classified as ambiguous/inconclusive using current recommendations of the American College for Medical Genetics/Association for Molecular Pathology [33]. Because genetic data are improved continuously, regular and careful reconsideration of genetic counseling and testing should take place, especially in those individuals/families in which a genetic cause of CHD is highly suspected but a genetic defect has not yet been identified [34]. The second main limitation is the large number of cases that currently remain without any associated alterations in the genes known to be associated with CHD. There are a limited number of genes known to cause CHD, partly due to the fact that the search for genes associated with CHD has focused mainly on exonic alterations, despite the fact that these regions only account for 2% of the whole human genome. Expanding this search to non-exonic regions could be key to identifying other genetic defects associated with this type of heart disease. The major challenge in studying genomic regions is the lack of reliable models to functionally validate identified alterations. Human induced-pluripotent stem cells derived from cardiomyocytes (hiPSC-CMs) have emerged as a potential tool to investigate genetic mechanisms of CHD using clinically relevant and patient-specific cardiac cells [35]. CRISPR/Cas9 genome editing tools have opened up new horizons to unravel the pathogenicity of the genetic variant and clarify pathophysiological mechanism involved in CHD. Despite this advance, results obtained should be carefully considered before translation to clinics.

## 4. Cardiac Malformations

More than fifty different types of cardiac malformations have been described; among these, atrial septal defect (ASD) is the most frequent, followed by ventricular septal defect (VSD) and persistent ductus arteriosus (PDA). Sometimes the same patient can manifest more than one CHD and this accumulation of malformations is usually associated with a worse prognosis. Clinically, CHD presents symptoms of different severity, many of which are mild forms that remit in the first months of life and do not impact adult life. This is the case in *foramen ovale*, an interatrial communications disorder that has a high incidence despite its low severity.

**Figure 1:**
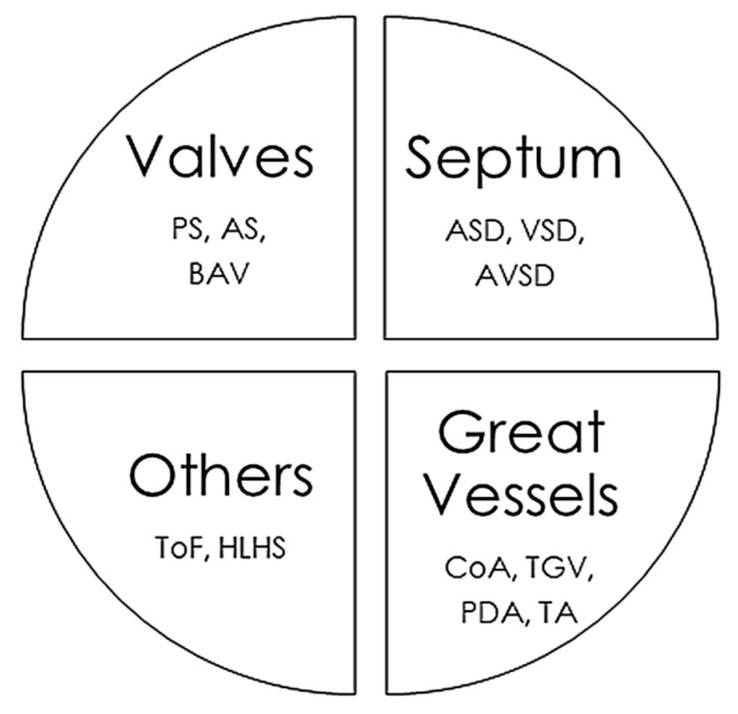
Main congenital heart defects and anatomic alterations. AS, Aortic valvular stenosis; ASD, Atrial septal defect; AVSD, Atrioventricular septal defect; BAV, Bicuspid aortic valve; CoA, Coarctation of the aorta; HLHS, Hypoplastic left heart syndrome, PDA, Persistent ductus arteriosus; PS, Pulmonary stenosis; ToF, Tetralogy of Fallot; TGV, Transposition of great vessels; TA, Common Artery Trunk or Truncus arteriosus; VSD, Ventricular septal defect.

The CHD classifications vary widely depending on the different factors considered, such as structural alterations, severity, frequency in the population, and whether or not they cause cyanosis. In this review, we have classified CHDs taking into account the associated structural alterations. Some pathologies are associated with multiple defects, so their classification is somewhat more complex and they could be placed in several groups simultaneously (Figure 1).

### 4.1. Short-Circuit Disturbances

In this type of defect, there is a direct communication between the right and left sides of the heart, which causes a mixture of blood from the pulmonary and systemic circulation. Among the pathologies associated with shunts, the most frequent are ASD, VSD, atrioventricular septal defect (AVSD), PDA, Common Artery Trunk (CTA) (at the level of the great arteries), and transposition of the great vessels (TGV). The severity of conditions associated with these pathologies depends on the size of the opening that allows both sides of the heart to communicate, leading to a left-to-right shunt based on the pressures, thus giving rise to pressure overload.

#### 4.1.1. Atrial Septal Defect

ASD is the most common CHD diagnosed in adults and represents 6% to 10% of all cardiac anomalies. It is more present in females. Based on the location of the defect in relation to the fossa ovalis and its size, we can differentiate three types of ASD: *ostium primum, ostium secundum,* and *sinus venosus* (5–10% of all ASD). The severity of ASD will depend on its size, presence of a left-to-right shunt and associated defects. Smaller defects do not require any treatment, while patients with more severe defects will require closure of the communication either by cardiac catheterization (of choice) or by surgical closure [36]. In 1998, four families showing different CHD were published [37]. All affected members carried an alteration in the *NKX2-5* gene, a transcription factor involved in correct heart development. It was the first gene associated with ASD. ASD occurs from spontaneous genetic alterations in genes such as *TBX5, NKX2-5, GATA4, NR2F2, ACVR1/ALK2,* and *CRELD1* (Table 1). A third of patients suffer from congenital syndromes (such as Down, Alagille, or Holt-Oram) which, among other defects, include ASD. Chromosome 5p mutation has been potentially associated with ASD, but a definite role should be confirmed.

#### 4.1.2. Ventricular Septal Defect

VSD represents 20% of patients in CHD studies and is slightly more common in females. Several types can be differentiated based on their location along the components of the ventricular septum [38], such as perimembranous (70% of VSD), subarterial (5–7%), muscular communication (5–20%) and channel-type communication (5–8%). VSD size and the magnitude of the left-to-right shunt determine the severity of this defect [39]. Depending on the severity of the communication defect between ventricles, surgery may be required, especially in the most severe cases [40]. Spontaneous closure of the defect occurs more frequently in those with a muscular build and can occur at any age, although it is more frequent in the first 6 months of life [41]. Maternal VDS leads to a 6–10% risk of recurrence while both parents’ presence of VSD is 2% [42,43]. In 1998, a report on four families showing different CHD was published [37]. All affected members carried an alteration in the *NKX2-5* gene, a transcription factor involved in correct heart development. This was the first gene associated with VSD. Sporadic pathogenic alterations associated with VSD are located mainly in the *NKX2-5, TBX5,* and *GATA4* genes [27,44]. These genes encode transcriptional factors involved in cardiac embryogenesis, essential for survival. Further studies have shown an interaction between *TBX5, GATA4*, and *NKX2.5,* suggesting that transcriptional activation may be responsible for septal defects [45]. Furthermore, VSD is the most frequent defect found in patients with Down syndrome (Table 1).

#### 4.1.3. Atrioventricular Septal Defect

An atrioventricular septal defect (AVSD, also called an AV canal) is quite rare (1:1300) with no gender differences, and accounts for 4–5% of heart defects diagnosed. AVSD can be classified into complete, partial (or incomplete) or transitional [46]. A common fact observed in AVSD babies is a serious heart alteration and around 50% of patients die during infancy. Surgery is usually needed and 95% of patients obtain a very successful result (15 years of survival) without significant complications [47]. The first gene associated with AVSD was *CRELD1,* reported in 2003 [48]. Nowadays, the cause of AVSD is not definitely known despite some genes having been potentially associated with the defect, such as *GJA1, GATA6, GATA4, CRELD1, NR2F2, TBX5* and *NKX2-5* [49]. All these genes codify for a key transcription factor involved in heart development except *CRELD1* (encode a protein involved in epidermal growth) and *GJA1* (encoding connexin 43, one of main proteins involved in intercellular communication between cells). In addition, AVSD can also occur in 15–20% of Down syndrome and with other types of CHD such as CoA or ToF.

#### 4.1.4. Persistent Ductus Arteriosus

PDA is usually identified in infants. In term neonates, the incidence is 1:2000 births, accounting for 5–10% of all CHD. In preterm neonates, it may range from 20–60% (1:10,000). PDA tends to affect girls more, although the reason is not yet known. In some restrictive CHD, such as TGA, it is necessary to keep the duct open after birth helping to form a mixture of oxygenated and non-oxygenated blood. In those newborns in whom closure does not occur spontaneously, drug treatment with indomethacin and ibuprofen is used and, in cases in which this option fails, a closure is resorted to by catheterization or surgery [50]. The PDA is closely related to preterm gestational age and low-weight preterm birth (45% of lower than 1750 g and 80% of 1200 g infants). Other relevant risk factors related to PDA are high altitude pregnancy. The presence of a sibling with PDA leads to a 3% possibility in the next offspring [2,51]. Most PDA is sporadic but it has been proposed as a multifactorial inheritance. In 2008, *TFAP2B* was suggested as the first defect for isolated nonsyndromic PDA [52]. This gene was usually related to Char Syndrome, a familial syndrome featured by PDA [53]. Inheritance of PDA is autosomal recessive with incomplete penetrance. Some of the genetic alterations that cause this pathology have been identified mainly in genes such as *ZEB2* (or *SMADIP1), TGFBR1/2, PTPN11* or *TBX1* (Table 1) [54]. The ZEB2 protein is a transcription factor that plays a role in the transforming growth factor β (TGFβ) signaling pathways that are essential during early fetal development. *TGFBR1/2* encodes a heteromeric complex protein with type II TGF-β receptors when bound to TGF-β, transducing the TGF-β signal from the cell surface to the cytoplasm (also associated with Loeys–Dietz aortic aneurysm syndrome). *PTPN11* encodes PTP protein, involved in cellular processes including cell growth, differentiation (also associated with Noonan syndrome and Leopard syndrome). Finally, *TBX1* encodes a key protein necessary for the normal development of large arteries (cases of 22q11.2 deletion syndrome contain *TBX1*). These genetic defects should be confirmed before definite association with PDA.

#### 4.1.5. Common Artery Trunk

CTA (also known as *common truncus arteriosus)* is a rare birth defect (1:10,000) that accounts for 4% of critical CHD. In patients with CTA, a single common blood vessel comes out of the heart instead of the main pulmonary artery and aorta vessels [55]. CTA is characterized by a VSD, a single truncal valve, and a common ventricular outflow tract. There are several different types of CTA, depending on how the arteries remain connected. CTA involves a single common valve (*truncal valve*) controlling blood flow out of the heart. Surgery is needed to repair CTA and is usually performed in the first few months of life with a long-term survival of 75% at 20 years [56]. Despite this fact, CTA still represents a significant challenge for cardiac surgeons and cardiologists regarding attempts to improve long-term outcomes as well as quality of life. The causes of CTA are unknown so far but it is frequently associated with 22q11 alterations [57]. In 2005 the first defect associated with CTA was published in the *NKX2-6* gene [58]. Nowadays, other variants in *NKX2-5, GATA6, TBX1*, and *ACTA2* have been suggested as potential causes of CTA. All these genes codify for a key transcription factor involved in heart development except *ACTA2* (encoding actin alpha 2, involved in contractile of smooth muscle). It is highly associated with DiGeorge syndrome (microdeletion 22q11. 2).

#### 4.1.6. Transposition of the Great Vessels

TGV represents 5–8% of all CHDs [59]. Cardiac surgery is the invariable therapeutic option for the survival of the newborn with this alteration. Surgical repairs include the atrial switch procedure, Senning or Mustard operation, the arterial procedure or the Rastelli operation [60]. In 2000, isolated mutations in *ZIC3* [61] and *CFC1* (human *CRYPTIC* gene) [62] were detected in patients with TGA. Other potentially pathogenic alterations have been identified in *GATA4, NKX2-5, MED13L,* and *PITX2* (Table 1). All these genes codify for a key transcription factor involved in heart development. This defect is not usually associated with the most frequent genetic syndromes, as was the case in previous malformations [63,64]. Among the potential related TGA genes, *NODAL* has emerged (involved in cell differentiation in early embryogenesis) which has been described in familial CHD, and *MEGF8* (which encodes a single pass membrane protein, known to participate in developmental regulation and cellular communication) associated with CHD in patients with Carpenter Syndrome subtype associated with lateralization abnormalities of the genetic alterations that cause this pathology. Despite the evidence, it has been suggested that the sole presence of genetic variants of *GDF1* (which has a role in left-right patterning and mesoderm induction during embryonic development) is not sufficient to cause TGA and requires other genetic anomalies [65].

### 4.2. Obstructive CHD

Obstructive CHDs include heart defects in which there is an obstruction of blood flow, including aortic coarctation (CoA), pulmonary stenosis (PS), bicuspid aortic valve (BAV), and aortic stenosis (AS).

#### 4.2.1. Aortic Coarctation

CoA occurs in 7% of patients with CHD, more frequently in males [66]. Depending on the degree of obstruction, the pathology will be more or less severe. Treatment is based on angioplasty or surgical repair [67]. It is widely related to Turner syndrome (4–14% of cases with Turner diagnosis), and with single rare variants, mainly in the *NOTCH1* gene (Table 1) [68,69]. It encodes a single-pass transmembrane receptor establishing an intercellular signaling pathway that plays a key role in development. Further studies are necessary with a large number of reported rare variants in order to confirm a definite association with CoA.

#### 4.2.2. Pulmonary Stenosis

PS occurs in 10–20% of all adult patients with CHD. The severity of the malformation depends on the degree of obstruction of blood flow, and symptoms may appear even in adulthood in the mildest cases. In these mildest cases, no treatment is necessary; in symptomatic cases or in moderate-severe stenosis, the treatment of choice is valvuloplasty by catheterization. The presence of PS is highly frequent in some genetic syndromes, mainly Noonan and Alagille syndrome. In 2003, the first rare alteration associated with non-syndromic PS [70] was reported in the *PTPN11* gene. In addition, single rare variants have been identified in genes such as *SOS1* and *JAG-1* (Table 1) [71]. SOS1 is a guanine nucleotide exchange factor (GEF) which interacts with RAS proteins, involved in the transduction of signals that control cell growth and differentiation (also associated with Noonan syndrome). Jagged1 interacts with receptors in the mammalian Notch signaling pathway, and establishes and regulates cell fate decisions in many organ systems during many developmental stages (associated with Alagille syndrome). All variants should be comprehensively analyzed and updated due to large number still having an ambiguous deleterious role.

#### 4.2.3. Bicuspid Aortic Valve

A bicuspid aortic valve (BAV) is the most common CHD (1:100) (Table 1) [72]. BAV is more common among males and often runs in families, hence first-degree relatives should be clinically assessed. Most people with a BAV are not affected by valve problems until they are adults, and 15% may not be affected until they are older adults [73]. Treatment may include aortic valve replacement, balloon valvuloplasty, aortic root, and ascending aorta surgery, or even aortic valve repair, although this is unusual [74]. Endocarditis can also occur in 2–5% of BAV, but the outcome of endocarditis tends to be worse than in normal valves. BAV often exists in babies with CoA. Nowadays, the cause of BAV is still unclear. BAV is inherited as an autosomal dominant trait with incomplete penetrance and variable expressivity due to a complex genetic architecture. Currently, only genetic alterations in *NOTCH1* have been definitively associated with BAV [75] despite rare variants in *MAT2A*, *ROBO4* and *ADAMTS19,* as well as common variants in *GATA4, 5, 6,* and *SMAD3, 4, 6* have been suggested as potential causes of disease [76]. These last genes codify a transcription factor involved in heart development but their role in BAV should be further analyzed.

#### 4.2.4. Aortic Valve Stenosis

The AS (or AoS) has an estimated prevalence of 1:12500 at birth. It is the most common valvular heart disease in the developed world, and affects about 2% of people who are over 65 years of age [77]. Symptoms often come on gradually and loss of consciousness typically occurs when standing or exercising. As with PS, the severity will be conditioned by the degree of involvement of the valve. Those patients with moderate-severe stenosis or symptoms should undergo catheterization or surgery. AS may be associated with Williams-Beuren syndrome, mainly caused by the *ELN* gene (Table 1) [78]. This gene encodes a protein that is one of the two components of elastic fibers. It was reported as the first gene associated with AS [79]. Other genes encoding transcription factors (*NR2F2, NOTCH1, SMAD6, TAB2, ROBO4*) have been suggested as a potential cause of AS, despite no definite role having been reported so far.

### 4.3. Tetralogy of Fallot

Tetralogy of Fallot (ToF) is the most common complex and cyanotic CHD, accounting for 7–10% of all CHD [80]. The prognosis is serious in the absence of treatment, which requires surgical intervention for total correction. In cases where this correction is successfully achieved, the prognosis is very favorable and a very acceptable quality of life is achieved. ToF is associated with a syndrome or chromosomal anomaly in 20% of cases. In most cases, the genetic cause that produces it is unknown [81], but it has been associated with multiple CNVs, one of the most common being the 22q11.2 deletion that affects cardiac transcription factor *TBX1* (Table 1) [82]. In 1998, a report on four families showing different CHD was published [37]. All affected members carried an alteration in the *NKX2-5* gene, a transcription factor involved in correct heart development. This was the first gene associated with ToF. Page et al. reported the importance of *NOTCH1* in non-syndromic ToF, as it is involved in organization of the outflow tract [83]. Besides, *FLT4* is a significant contributor to ToF whose product, VEGFR3, is present in the development of the great vessels. Other genes, mainly encoding transcription factors, have been suggested as potential cause (*ZFPM1/FOG1, CAMTA2, DLX6*) despite further studies being necessary to conclude a definite role in ToF.

### 4.4. Hypoplastic Left Heart Syndrome

Hypoplastic left heart syndrome (HLHS) is a rare birth defect (1:7000) accounting for 3% of all infants born with CHD [84]. It is one of the more complex and severe CHDs, with an atypical and diverse molecular pathophysiology, responsible for nearly 25% of cardiac deaths during the first two days of life. Therefore, multiple surgeries are needed in the first hours/days of life to increase blood flow to the body and bypass the poorly functioning left side of the heart. After various complex surgeries, only 48% of patients survive up to 20 years thereafter. In the most severe forms of the disease, a heart transplant is required for survival [85]. More than 25% of patients with HLHS had one or more relatives with a CHD, most commonly BAV. A genetic association of HLHS with other left-sided obstructive lesions (BAV, CoA, AVS) has been reported. In 2003 the first definite association with HLHS caused by rare variants in the *GJA1* gene, encoding connexin43 protein [86], was reported. Currently, potential genes associated with HLHS include *NKX2-5,* and *NOTCH1,* but a definite role should be analyzed in a larger number of rare variants before clinical translation [87].

## 5. Syndromes with Congenital Cardiac Alteration

**Figure 2:**
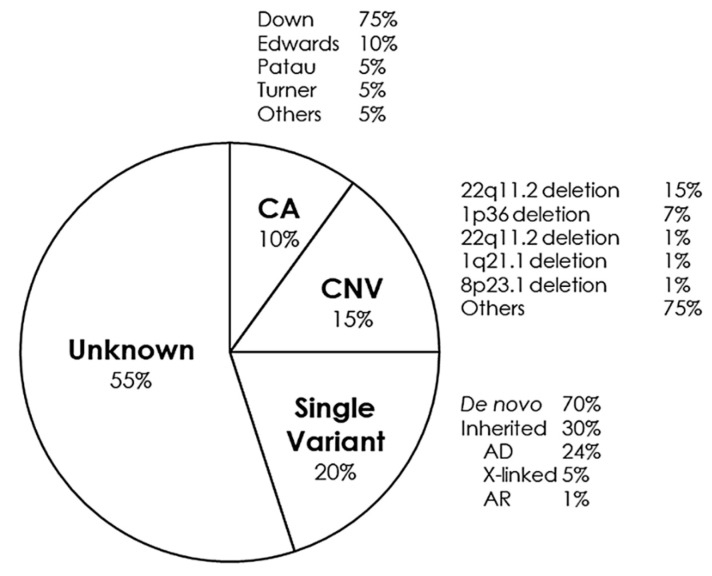
Genetic distribution of congenital heart defects. AD, Autosomal Dominant; AR, Autosomal Recessive; CA, Chromosomic Alterations; CNV, Copy Number Variants.

CHDs can occur in isolation, but approximately a quarter are associated with syndromes of genetic origin that lead to other extracardiac malformations. CHD can be found in 35–50% of children born with trisomy 21 (Down syndrome), in 60–80% of children born with trisomy 13 and 18 (Patau and Edwards syndrome, respectively) and in 33% of girls with monosomy X (Turner syndrome) [88]. Some of the main syndromes that occur with CHD are detailed below (Figure 2).

### 5.1. Down Syndrome

The incidence of trisomy 21 is around 1:1000 pregnancies. In nearly 95% of cases, trisomy occurs due to an additional independent copy of chromosome 21 caused by an accidental non-disjunction during meiosis. The recurrence risk is 1% until the age of 40 years. It is characterized by slanting eyes, protruding tongue, variable cognitive dysfunction, muscular hypotonia, and joint laxity. It can be accompanied by heart, gastrointestinal, and endocrine defects. Down syndrome was genetically characterized by Lejeune in 1959 [25,89,90]. More than 60 years later, in almost 50% of cases, heart defects are observed, mainly VSD, which is seen in 43% of cases, and to a lesser extent secumdum-type ASD, seen in 42% of cases, as well as PAD, ToF, and AVSD (Table 1) [91,92].

### 5.2. Edwards Syndrome

Trisomy 18 has an estimated incidence of 1/6000–1/8000 pregnancies. In utero death occurs in nearly 90% of affected fetuses. Most cases are associated with free trisomy 18. The risk of recurrence of trisomy is around 1%. However, in families in which trisomy 18 is caused by translocation, the recurrence risk is higher if one of the parents is a carrier of a balanced translocation. It presents a very characteristic phenotype in most cases, with ocular malformations together with digestive, urinary, and cardiac abnormalities. In 1960, Edwards reported a new trisomic syndrome [93]. Nowadays, nearly 80% of born babies with this trisomy present a CHD [94]. The most frequent cardiac malformations in this syndrome are VSD, valve dysplasia, and ToF. Other CHDs reported in Edward’s syndrome are AVSD, double-outlet right ventricle, and CoA (Table 1) [95].

### 5.3. Patau Syndrome

Trisomy 13 has an estimated incidence of 1/8000–1/15,000 pregnancies. Free trisomy 13 is found in around 75% of cases. In 20% of cases, trisomy 13 is associated with a Robertsonian translocation. The risk of recurrence is around 1%. However, in families in which trisomy 13 is associated with translocation, the risk of recurrence is higher. It is characterized by brain malformations, severe psychomotor development, facial dysmorphism, ocular abnormalities, as well as cardiac and urogenital malformations. CHDs are present in 51–64% of live born babies with this trisomy, and was first reported by Patau et al. in 1960 [96,97]. Cardiac malformations include ASD, VSD, ToF, nodular valvular dysplasia, and conotruncal CHD (Table 1) [98].

### 5.4. Turner Syndrome, Ullrich-Turner Syndrome, or 45,X Syndrome

Partial or complete absence of an X chromosome has an incidence in 1 in every 5000 live births (1:2500 female births). Its prevalence is influenced by the presence of the X chromosome or whether it is structurally abnormal [99]. The most important clinical features are neck webbing, horseshoe kidney, short height and infertility. Variable-onset ovarian failure is common in this syndrome and, although less frequently, bone abnormalities, lymphedema, deafness, and gastrointestinal, thyroid, and cardiovascular involvement may occur. In up to 30% of cases, affects mainly in the left-sided heart, such as hypoplastic left heart syndrome (HLHS), bicuspid aortic valve (BAV), CoA, and AS, are identified (Table 1) [100]. It was clinically reported and first identified by Turner in 1938 [101], but the genetic basis was reported by Ford et al. in 1959 [102,103,104].

### 5.5. DiGeorge Syndrome or 22q11.2 Deletion (Takao Syndrome, Velocardiofacial Syndrome, Cayler Cardiofacial Syndrome)

DiGeorge syndrome is one of the most frequent syndromes (1 in 6000). In a large number of patients, it occurs de novo. This syndrome is associated with heterogeneous clinical pictures, even within the same family. Generally, patients present alterations such as immunodeficiency, hypocalcemia, hypoparathyroidism, characteristically abnormal facial features, impaired neurological development and cardiac malformations [105]. The latter can be identified in up to 70% of cases, and consist mainly of cono-truncal development impairment such as anomalies of the aortic arch (right aortic arch, double or interrupted type B aortic arch), misaligned VSD, HLHS, TGV, ASD, pulmonary atresia or PS, ToF, and CTA (Table 1). This syndrome was clinically reported by DiGeorge et al. in 1968 [106] and genetically characterized in 1981 [107]. The microdeletion causing this syndrome affects genes such as *TBX1*, which is involved in the process of heart formation. *TBX1* deficiency produces alterations in the ventriculoarterial connection outflow tract. Moreover, *TBX1* gene dosage may influence the physiological development of the heart [108].

### 5.6. Trisomy 22q11.2 or 22q11.2 Duplication Syndrome

This syndrome is characterized by a phenotypic picture similar to 22q11.2 deletion (autosomal dominant) (Table 1). It was firstly reported in 1999 [109]. Nowadays, nearly 90% of cases are caused by de novo alterations. Cardiac malformations are less frequent than in the deletion of the same region of the chromosome, but septal defects and obstructive lesions in the left ventricle may appear, associated with neurological and growth delays [110].

### 5.7. 1q21.1 Deletion and Duplication

These alterations give rise to moderate cognitive dysfunction, microcephaly, and very frequent CHD. They were first reported in 2008 by Mefford et al. [111]. Among these, obstructions on the left side (40%), septal defects (27%), and conotruncal anomalies (20%) are the main defects. It is known that 1q21.1 detection syndrome affects the *GJA5* gene, which is involved in the heart defect, but no underlying mechanism has been proposed. Duplication is much less common, but includes the *GJA5* gene, associated with ToF and development of malignant arrhythmias with risk of sudden death. The former may be related to the connexin-40 gap union protein encoded by *GJA5* (Table 1) [112].

### 5.8. Subtelomeric 1p36 Deletion or 1p36 Deletion Syndrome

This syndrome (1:5000–1:10,000) is the second most common type of microdeletion. It is characterized by cognitive dysfunction, epilepsy, dysmorphic features, and metabolic and neuromuscular disorders. In 50% of cases, it is possible to find cardiac malformations, the most frequent being the non-compaction cardiomyopathy, ASD, VSD, ToF, PDA, and Epstein’s anomaly. In 1997, the deletion of chromosome 1p36 was reported in a syndrome with multiple congenital anomalies and mental retardation [113]. It is also associated with rare alterations in the *PRDM16* gene (Table 1) [114]. The protein encoded by this gene is a zinc finger transcription factor and acts as a transcription coregulator that controls the development of brown adipocytes in brown adipose tissue.

### 5.9. Monosomy 8p23.1 or 8p23.1 Deletion

This deletion gives rise to cardiac malformations in more than 90% of cases. These alterations range from isolated defects, such as VAS and ASD, to complex defects such as ToF and HLHS. The first case of an 8p deletion was described by Lubs and Lubs in 1973 [115]. Nowadays, we know that the origin of these malformations is the absence of the transcription factor *GATA4*, which plays a fundamental role in heart development (Table 1). Other transcription factors encoded by *NKX-2, TBX5, ZFPM2 (FOG2), SMAD4,* and *HAND2* are related to *GATA4* in heart embryogenesis. This crossover may be explained by epistatic effects leading to complex CHD phenotypes observed in a subgroup of 8p23 deletion patients [116,117].

### 5.10. Kleefstra Syndrome

The prevalence of this syndrome is currently unknown but it is considered an ultra-rare disease (<1 in 100,000), caused by a microdeletion in the 9q34.3 region or by single rare variants in the *EHMT1* gene (following an autosomal dominant pattern of inheritance) involved in methyltransferase activity [118]. A similar phenotype is reported in patients with pathogenic variants in the *KMT2C* gene. It encodes a nuclear protein which possesses histone methylation activity and is involved in transcriptional coactivation. Those affected show cognitive dysfunction and typical facial characteristics (ocular hypertelorism, thick and everted lower lip, macroglossia, and anteverted nostrils), and cardiac malformations are present in 40% of patients (VSD, ASD, CoA, PS, or ToF) (Table 1). Patients with associated *COL5A1* and *NOTCH1* mutations are more likely to have aortic valve anomalies and pulmonary defects. Yatsenko et al. described a family with Kleefstra syndrome with mutations in *CACNA1B* but not *EHMT* [119]. These last studies should be confirmed by additional reports in order to establish a definite deleterious role.

### 5.11. Wolf-Hirschhorn Syndrome or Telomeric Deletion 4p

The prevalence of Wolf-Hirschhorn syndrome is estimated at 1 in every 50,000 live births. This syndrome is caused by loss of the distal portion of the 4p region and is characterized by facial alterations, growth delays, and neurological abnormalities, as well as seizures. It was reported in 1965 [120,121]. In 50% of cases, cardiac malformations are observed, mainly ASD, VSD, and PDA (Table 1). Most cases are sporadic, but an unbalanced translocation may be inherited from a parent with a balanced rearrangement. The main gene affected by the deletion is *WHSC1*, which is involved in the formation of the cardiac septum [122].

### 5.12. Williams-Beuren Syndrome

Williams-Beuren syndrome is a rare entity (1:7500) caused by a deletion of 1.5–1.8 Mbp in the 7q11.23 region, affecting 28 genes. It is characterized by infantile hypercalcemia, skeletal and kidney abnormalities, cognitive deficits and a characteristic personality, as well as cardiovascular abnormalities. Cardiac alterations occur in almost 75% of cases, with the most common being supravalvular AS and PS, among others, including atypical cardiac defects such as ASD, VSD, and ToF. It was firstly reported in 1961 by Williams et al. [123]. Beuren et al. described in 1962 a similar syndrome with additional features [124]. High clinical variability has been described, as deletion of different genes gives rise to different clinical manifestations. Deficiency of the *ELN* gene (Table 1), encoding elastin, is associated with supravalvular AS, with an autosomal dominant pattern of inheritance [125]. However, *ELN* deficiency does not explain the atypical cardiac defects which have been associated with changes in *BAZ1B* expression caused by epigenetic effects [126]. This gene encodes a member of the bromodomain protein family involved in chromatin-dependent regulation of transcription, but additional studies are necessary to confirm the pathogenic association.

### 5.13. Noonan Syndrome

Noonan syndrome is rare (1:2500) despite being one of the most frequent genetic syndromes associated with cardiac malformation. The clinical characteristics of this syndrome are short stature, facial dysmorphic characteristics, hematological, dermatological and skeletal alterations, and cognitive dysfunction, among others. Cardiac malformation occurs in 60–90% of cases and the most frequent anomalies are PS, associated with a dysplastic pulmonary valve, AVSD, ASD, ToF, CoA, and 20% of hypertrophic cardiomyopathy cases.

It was firstly reported by Noonan et al. in 1968 [127]. Currently, pathogenic variants in *PTPN11* are seen in 50% of cases, *SOS1* variants are seen in 15% of cases, and *RAF1*, *RIT1, KRAS, SHOC2, NRAS, SOS2*, *BRAF,* and *LZTR1* variants are also observed (Table 1). Inheritance is autosomal dominant, except for *LZTR1,* which can be either dominant or recessive [128]. This gene encodes a protein of the Golgi network, helping to stabilize this key cell organelle. Different genetic defects may be related to the cardiac presentation; the *SOS1* defect is more related to PS, ASD is relatively rare in patients with a *PTPN11* mutation, and *RAF1* mutations are associated with a high prevalence of hypertrophic cardiomyopathy [129,130].

### 5.14. Costello Syndrome or Faciocutaneoskeletal Syndrome

Costello syndrome is an ultra-rare entity (1:300,000) characterized by coarse facial features, deep creases of the palms and soles, hypotonia, feeding problems, higher risk for malignancies, and cognitive dysfunction. ASD, CoA, or ToF may be present in this syndrome. Costello syndrome shows significant clinical overlap with Noonan syndrome and cardiofaciocutaneous syndrome, as well as RASopathies such as Beckwith-Wiedemann, Noonan syndrome with multiple lentiginous (formerly known as LEOPARD syndrome) and Simpson-Golabi-Behmel syndromes. This syndrome was firstly reported by Costello et al. in 1977 [131]. Pathogenic variants in *HRAS* have been described to follow an autosomal dominant pattern of inheritance (Table 1) [132,133]. HRas is a small G protein in the Ras-MAPK subfamily, involved in regulating cell division [134].

### 5.15. Leopard Syndrome or Cardiomyopathic Lentiginosis

Leopard syndrome is an ultra-rare entity (1:300,000) characterized by multiple lentigines of the face, back and upper trunk and characteristic facial features. Mitral valve defects, ASD, CoA, and ToF may be present in this syndrome. Patients may have an initial diagnosis of Noonan syndrome and genetic testing may be useful to distinguish between overlapping syndromes. LEOPARD is an acronym for the manifestations of this syndrome as listed by Gorlin et al. in 1969 [135]. Pathogenic variants in *PTPN11, RAF1, MAP2K1,* and *BRAF* follow an autosomal dominant pattern of inheritance (Table 1) [136].

### 5.16. Holt-Oram Syndrome or Heart-Hand Syndrome Type 1

This ultra-rare entity (1:100,000) is characterized by a wide spectrum of malformations in the upper extremities together with cardiac alterations, mainly ASD, and sometimes VSD as well as defects in the electrical conduction of the heart and Epstein’s anomaly. It was first reported in 1960 [137]. To date, Holt-Oram syndrome is only caused by pathogenic rare alterations in the *TBX5* gene in almost 80% of cases (autosomal dominant inheritance) (Table 1). A large number of the associated genetic variants occur de novo [138,139].

### 5.17. Adams-Oliver Syndrome or Limb, Scalp, and Skull Defects

This is an ultra-rare entity (<1:100,000) characterized by asymmetric malformations of the fingers and toes, as well as defects in the arms and legs. In 20% of cases, scalp defects are also identified. Congenital heart malformations are only present in 9% of patients and are usually ASD, VSD, ToF, AS, and HLHS. This syndrome was reported for the first time in 1945 [140]. It occurs due to pathogenic rare variants in genes such as *NOTCH1, ARHGAP31* (encoding GAP, a Rho family GTPases involved in regulation of intracellular pathways)*, DOCK6* (a member of the DOCK-C subfamily of the DOCK family of guanine nucleotide exchange factors which function as activators of small G proteins)*, RBPJ, DLL4* and *EOGT* (all three involved in the Notch signaling pathway) (Table 1). Most cases are transmitted as an autosomal dominant trait, but *DOCK6* and *EOGT* follow an autosomal recessive transmission with familial or sporadic occurrence [141].

### 5.18. Alagille Syndrome or Alagille-Watson Syndrome or Arteriohepatic Dysplasia

This rare entity (1:70,000) is characterized by multisystem involvement (mainly heart, liver, eyes, and skeleton). Cardiac involvement is present in 90% of cases, and the cardiac alteration may be PS, VSD, ASD, PDA, or ToF. Between 3–7% of patients have a 20p12 microdeletion. This syndrome was firstly reported by Watson et al. in 1973 [142], and clinically completed by Alagille et al. two years later [143]. Pathogenic rare variants are usually identified in the *JAG1* gene (it is one of five ligands for NOTCH receptors and helps to determine cellular fate during many developmental phases), although other minor genes such as *NOTCH2* (which establishes an intercellular signaling pathway that plays a key role in development) are also affected (Table 1). The inheritance is autosomal dominant, but reduced penetrance (up to 50% of cases) and somatic mosaicism (~8%) are common [14,144].

### 5.19. Kabuki Syndrome or Niikawa-Kuroki Syndrome

This is a rare syndrome (1:32,000) characterized by a highly variable clinical spectrum. It usually involves developmental delay, facial alterations, and various cardiac alterations and was firstly reported by Niikawa et al. in 1981 [145]. It is due mainly to pathogenic rare alterations in *MLL2* and *KMT2D* (up to 80% of cases) (autosomal dominant inheritance), and *KDM6A* (X-dominant inheritance). KMTD2 is a large protein which co-localizes with transcription factors, being essential for cell differentiation and embryonic development. The *KDM6A* gene encodes lysine-specific demethylase 6A, also called ubiquitously transcribed tetratricopeptide repeat X chromosome (UTX), involved in regulation of iron reactions such as histone and DNA methylation. This rare syndrome is associated with ASD, VSD, HLHS, ToF, AS, and Epstein’s anomaly (Table 1) [146,147,148,149].

### 5.20. CHARGE Syndrome

This is an ultrarare syndrome (1:100,000) characterized by coloboma, cardiac malformation, choanal atresia, delayed growth and development, genital hypoplasia, hearing abnormalities, or deafness (acronym of CHARGE). The first descriptions of this syndrome were provided in 1979 by Hall [150], and Hittner et al. [151]. Most of the associated rare pathogenic variants are located in the *CHD7* gene, which is mutated in 70% of cases (autosomal dominant inheritance). The protein belongs to a larger group of ATP-dependent chromatin remodeling complexes assisting in chromatin access and nucleosome editing. At the heart level, the most common defects are ToF, HLHS, AVSD, VSD, PDA, TA, CoA, and Epstein’s anomaly (Table 1) [152].

### 5.21. Koolen-De Vries Syndrome

This is a rare syndrome (1:50,000) characterized by severe intellectual deficit, hypotonia, epileptic seizures, and facial dysmorphia. In around 35% of cases, it is also possible to find cardiac malformations, mainly VSD, ASD and PS (Table 1) [153,154,155]. It was reported in 1993 by Koolen et al. [154] and is caused by 17q21.31 deletion or rare pathogenic alteration in the *KANSL1* gene (autosomal dominant inheritance). This gene (also called *KAT8*) encodes a nuclear protein that is a subunit of two protein complexes involved with histone acetylation. All deletions identified to date are de novo.

### 5.22. Jacobsen Syndrome or Telomeric Deletion 11q

This is a rare disorder (1:50,000) characterized by craniofacial dysmorphism, congenital cardiac alterations (VSD, HLHS, AVSD, PDA, TGA and AS), intellectual disability, Paris Trousseau bleeding disorder, structural kidney defects and immunodeficiency (Table 1). The disorder was first observed by Jacobsen et al. in 1973 [156]. It is caused by deletions in the long arm of chromosome 11 (11q), and about 90% of cases are de novo [157].

### 5.23. Char Syndrome or Patent Ductus Arteriosus with Facial Dysmorphism and Abnormal Fifth Digits

This is an ultra-rare syndrome (<1:1,000,000) characterized by facial dysmorphism and hand anomalies (aplasia or hypoplasia of the middle phalanges of the fifth fingers). Additional features associated with Char syndrome include heart defects such as VSD and PDA. It was reported in 1993 [158]. So far, causal rare variants have only been only identified in the *TFAP2B* gene (autosomal dominant inheritance) (Table 1) [159]. This gene encodes AP-2 beta protein, involved in cell proliferation during embryonic development.

### 5.24. Myhre Syndrome or Facial Dysmorphism-Intellectual Disability-Short Stature-Deafness Syndrome

This is an ultra-rare entity (<1:1,000,000) characterized by short stature, distinctive facial dysmorphism, brachydactyly, stiff and thick skin, muscular pseudohypertrophy, restricted joint mobility, hearing loss, and variable cognitive dysfunction. Heart alterations are commonly observed, such as ToF, CoA and AS (Table 1). Myhre Syndrome was first reported in 1981 [160] and is due to heterozygous pathogenic variant in the *SMAD4* gene, following an autosomal dominant pattern of inheritance, despite most cases being de novo [161]. The protein, also named DPC4, regulates critical processes during embryo development, tissue homeostasis, regeneration, and immune regulation.

### 5.25. Ellis Van Creveld Syndrome or Chondroectodermal Dysplasia

This is an ultra-rare entity (<1:1,000,000) characterized by short ribs, polydactyly, growth retardation, and ectodermal as well as heart defects in nearly 60% of cases, such as ASD, HLHS, PDA and VSD [162]. This syndrome was reported in 1964 [163]. It is caused by a pathogenic variant (primarily frameshift resulting in a nonsense codon) in *EVC* and *EVC2* genes, following an autosomal recessive pattern of inheritance (Table 1) [164]. Both genes encode proteins containing a leucine zipper and a transmembrane domain, and act as mediators for signaling.

## 6. Conclusions

CHDs are defects that occur during embryonic development of the heart. The etiology is multifactorial but genetic alterations are the main cause. Advances in clinical diagnosis and treatment have allowed a high percentage of patients to survive and reach adulthood with a good quality of life. Use of novel genetic technologies has made it possible to identify a multitude of genetic defects responsible for this type of malformation. This has increased the number of cases with a genetic diagnosis in the last ten years and has allowed early identification of cases, as well as offering adequate genetic counseling for affected families who wish to have offspring. Genetic diagnosis is heavily dependent on accurate clinical diagnosis. An early conclusive diagnosis as well as the adoption of personalized therapeutic measures is crucial in the favorable evolution of the newborn.

## Figures and Tables

**Table 1:** Main congenital heart defects.

Pathology	Frequency	Associated Genes
Aortic valve stenosis	1:12,500	*ELN, NR2F2, NOTCH1, SMAD6, TAB2, ROBO4*
Atrial septal defect or Interauricular communication	1:1000	*MYH6, ACTC1, GATA4, TBX20, TLL1, CITED2, GATA6, TBX5, TBX6, NKX2-5, GATA4, NR2F2, ACVR1/ALK2, CRELD1*
Atrioventricular septal defect	1:1300	*GJA1, GATA6, GATA4, CRELD1, NR2F2, TBX5, NKX2-5*
Bicuspid aortic valve	1:100	*NOTCH1*
Coarctation of the aorta	1:7000	*NOTCH1, NR2F1, SMAD6, NKX2-5, NKX2-6, GATA6, TBX1*
Hypoplastic left heart syndrome	1:7000	*GJA1, NKX2-5, PCDHA13, NOTCH1*
Persistent ductus arteriosus	1:11,000	*ACTA2, PRDM6, R187, TFAB2B, SMADIP1, TGFBR1/2, PTPN11, TBX1, MYH11*
Supravalvular aortic and pulmonary artery stenosis	1:2500	*PTPN11, SOS1, JAG1, ELN, GATA4*
Tetralogy of Fallot	1:2000	*JAG1, TBX1, NKX2-5, NKX2-6, GATA4, GATA5, GATA6, NR2F2, ZFPM2/FOG2, NOTCH1, TAB2*
Transposition of great vessels	1: 3000	*GATA4, NKX2-5, MED13L, PITX2*
Common artery trunk	1:10,000	*NKX2-5, NKX2-6, GATA6, TBX1, ACTA2, R187*
Ventricular septal defect or interventricular communication	1:300	*GATA4, CITED2, ETS1, TBX5, TBX1, NKX2-5*

## References

[1] Campbell M. (1949). Genetic and environmental factors in congenital heart disease. Q. J. Med..

[2] Nora J.J. (1968). Multifactorial inheritance hypothesis for the etiology of congenital heart diseases. The genetic-environmental interaction. Circulation.

[3] Shi H., O’Reilly V.C., Moreau J.L., Bewes T.R., Yam M.X., Chapman B.E., Grieve S.M., Stocker R., Graham R.M., Chapman G. (2016). Gestational stress induces the unfolded protein response, resulting in heart defects. Development.

[4] Vecoli C., Pulignani S., Foffa I., Andreassi M.G. (2014). Congenital heart disease. the crossroads of genetics, epigenetics and environment. Curr. Genomics.

[5] De Backer J., Callewaert B., Muino Mosquera L. (2020). Genetics in congenital heart disease. Are we ready for it?. Rev. Esp. Cardiol..

[6] Leirgul E., Fomina T., Brodwall K., Greve G., Holmstrom H., Vollset S.E., Tell G.S., Oyen N. (2014). Birth prevalence of congenital heart defects in Norway 1994-2009—A nationwide study. Am. Heart J..

[7] Bernier P.L., Stefanescu A., Samoukovic G., Tchervenkov C.I. (2010). The challenge of congenital heart disease worldwide. epidemiologic and demographic facts. Semin. Thorac. Cardiovasc. Surg. Pediatr. Card. Surg. Annu..

[8] Perez-Lescure Picarzo J., Mosquera Gonzalez M., Latasa Zamalloa P., Crespo Marcos D. (2018). Incidence and evolution of congenital heart disease in Spain from 2003 until 2012. An. Pediatr..

[9] Jacobs J.P., Mayer J.E., Pasquali S.K., Hill K.D., Overman D.M., St Louis J.D., Kumar S.R., Backer C.L., Tweddell J.S., Dearani J.A. (2019). The Society of Thoracic Surgeons Congenital Heart Surgery database. 2019 update on outcomes and quality. Ann. Thorac. Surg..

[10] Jacobs M.L., Jacobs J.P., Hill K.D., O’Brien S.M., Pasquali S.K., Vener D., Kumar S.R., Chiswell K., St Louis J.D., Mayer J.E. (2019). The Society of Thoracic Surgeons Congenital Heart Surgery database. 2019 update on research. Ann. Thorac. Surg..

[11] Raissadati A., Nieminen H., Jokinen E., Sairanen H. (2015). Progress in late results among pediatric cardiac surgery patients. a population-based 6-decade study with 98% follow-up. Circulation.

[12] Gilboa S.M., Mai C.T., Shapiro-Mendoza C.K., Cragan J.D., Moore C.A., Meaney-Delman D.M., Jamieson D.J., Honein M.A., Boyle C.A. (2017). Population-based pregnancy and birth defects surveillance in the era of Zika virus. Birth Defects Res..

[13] Pierpont M.E., Brueckner M., Chung W.K., Garg V., Lacro R.V., McGuire A.L., Mital S., Priest J.R., Pu W.T., Roberts A. (2018). Genetic basis for congenital heart disease. revisited. A scientific statement from the American Heart Association. Circulation.

[14] Zaidi S., Brueckner M. (2017). Genetics and genomics of congenital heart disease. Circ. Res..

[15] Nees S.N., Chung W.K. (2020). The genetics of isolated congenital heart disease. Am. J. Med. Genet. C Semin. Med. Genet..

[16] Halloran K.H., Hsia E., Rosenberg L.E. (1976). Genetic counseling for congenital heart disease. J. Pediatr..

[17] Blue G.M., Kasparian N.A., Sholler G.F., Kirk E.P., Winlaw D.S. (2015). Genetic counselling in parents of children with congenital heart disease significantly improves knowledge about causation and enhances psychosocial functioning. Int. J. Cardiol..

[18] European Society of Human Group (2010). Statement of the ESHG on direct-to-consumer genetic testing for health-related purposes. Eur. J. Hum. Genet..

[19] Zaidi S., Choi M., Wakimoto H., Ma L., Jiang J., Overton J.D., Romano-Adesman A., Bjornson R.D., Breitbart R.E., Brown K.K. (2013). De novo mutations in histone-modifying genes in congenital heart disease. Nature.

[20] Sifrim A., Hitz M.P., Wilsdon A., Breckpot J., Turki S.H., Thienpont B., McRae J., Fitzgerald T.W., Singh T., Swaminathan G.J. (2016). Distinct genetic architectures for syndromic and nonsyndromic congenital heart defects identified by exome sequencing. Nat. Genet..

[21] Oyen N., Poulsen G., Boyd H.A., Wohlfahrt J., Jensen P.K., Melbye M. (2009). Recurrence of congenital heart defects in families. Circulation.

[22] Peyvandi S., Ingall E., Woyciechowski S., Garbarini J., Mitchell L.E., Goldmuntz E. (2014). Risk of congenital heart disease in relatives of probands with conotruncal cardiac defects. An evaluation of 1620 families. Am. J. Med Genet. Part A.

[23] Wen S.W., Miao Q., Taljaard M., Lougheed J., Gaudet L., Davies M., Lanes A., Leader A., Corsi D.J., Sprague A.E. (2020). Associations of assisted reproductive technology and twin pregnancy with risk of congenital heart defects. JAMA Pediatr..

[24] Giorgione V., Parazzini F., Fesslova V., Cipriani S., Candiani M., Inversetti A., Sigismondi C., Tiberio F., Cavoretto P. (2018). Congenital heart defects in IVF/ICSI pregnancy. systematic review and meta-analysis. Ultrasound Obstet. Gynecol..

[25] Lejeune J., Turpin R., Gautier M. (1959). Chromosomic diagnosis of mongolism. Arch. Fr. Pediatr..

[26] Ahrens-Nicklas R.C., Khan S., Garbarini J., Woyciechowski S., D’Alessandro L., Zackai E.H., Deardorff M.A., Goldmuntz E. (2016). Utility of genetic evaluation in infants with congenital heart defects admitted to the cardiac intensive care unit. Am. J. Med. Genet. Part A.

[27] Lalani S.R., Belmont J.W. (2014). Genetic basis of congenital cardiovascular malformations. Eur. J. Med. Genet..

[28] Vetrini F., D’Alessandro L.C., Akdemir Z.C., Braxton A., Azamian M.S., Eldomery M.K., Miller K., Kois C., Sack V., Shur N. (2016). Bi-allelic Mutations in PKD1L1 are associated with laterality defects in humans. Am. J. Hum. Genet..

[29] Homsy J., Zaidi S., Shen Y., Ware J.S., Samocha K.E., Karczewski K.J., DePalma S.R., McKean D., Wakimoto H., Gorham J. (2015). De novo mutations in congenital heart disease with neurodevelopmental and other congenital anomalies. Science.

[30] Blue G.M., Kirk E.P., Giannoulatou E., Sholler G.F., Dunwoodie S.L., Harvey R.P., Winlaw D.S. (2017). Advances in the Genetics of Congenital Heart Disease. A Clinician’s Guide. J. Am. Coll. Cardiol..

[31] Mone F., Stott B.K., Hamilton S., Seale A.N., Quinlan-Jones E., Allen S., Hurles M.E., McMullan D.J., Maher E.R., Kilby M.D. (2021). The Diagnostic yield of prenatal genetic technologies in congenital heart disease. A prospective cohort study. Fetal Diagn. Ther..

[32] Mone F., Eberhardt R.Y., Morris R.K., Hurles M.E., McMullan D.J., Maher E.R., Lord J., Chitty L.S., Giordano J.L., Wapner R.J. (2021). Congenital heart disease and the Diagnostic yield with Exome sequencing (CODE) study. prospective cohort study and systematic review. Ultrasound Obstet. Gynecol..

[33] Richards S., Aziz N., Bale S., Bick D., Das S., Gastier-Foster J., Grody W.W., Hegde M., Lyon E., Spector E. (2015). Standards and guidelines for the interpretation of sequence variants. a joint consensus recommendation of the American College of Medical Genetics and Genomics and the Association for Molecular Pathology. Genet. Med..

[34] Bowdin S., Gilbert A., Bedoukian E., Carew C., Adam M.P., Belmont J., Bernhardt B., Biesecker L., Bjornsson H.T., Blitzer M. (2016). Recommendations for the integration of genomics into clinical practice. Genet. Med..

[35] Lin H., McBride K.L., Garg V., Zhao M.T. (2021). Decoding genetics of congenital heart disease using patient-derived induced pluripotent stem cells (iPSCs). Front Cell Dev. Biol..

[36] Salehian O., Horlick E., Schwerzmann M., Haberer K., McLaughlin P., Siu S.C., Webb G., Therrien J. (2005). Improvements in cardiac form and function after transcatheter closure of secundum atrial septal defects. J. Am. Coll. Cardiol..

[37] Schott J.J., Benson D.W., Basson C.T., Pease W., Silberbach G.M., Moak J.P., Maron B.J., Seidman C.E., Seidman J.G. (1998). Congenital heart disease caused by mutations in the transcription factor NKX2-5. Science.

[38] Soto B., Becker A.E., Moulaert A.J., Lie J.T., Anderson R.H. (1980). Classification of ventricular septal defects. Br. Heart J..

[39] Minette M.S., Sahn D.J. (2006). Ventricular septal defects. Circulation.

[40] Li C.S., Lu Z., Song X.R., Yan Z.Y. (2019). Hybrid procedure for treating adult congenital heart disease with valvular heart disease in two patients. J. Cardiothorac. Surg..

[41] Erol O., Sevket O., Keskin S., Yazicioglu H.F., Gul A. (2014). Natural history of prenatal isolated muscular ventricular septal defects. J. Turk. Ger. Gynecol. Assoc..

[42] Nora J.J., Nora A.H. (1988). Familial risk of congenital heart defect. Am. J. Med. Genet..

[43] Nora J.J., Nora A.H. (1988). Update on counseling the family with a first-degree relative with a congenital heart defect. Am. J. Med. Genet..

[44] Chowdhury R., Ashraf H., Melanson M., Tanada Y., Nguyen M., Silberbach M., Wakimoto H., Benson D.W., Anderson R.H., Kasahara H. (2015). Mouse model of human congenital heart disease: Progressive atrioventricular block induced by a heterozygous Nkx2-5 homeodomain missense mutation. Circ. Arrhythm. Electrophysiol..

[45] Garg V., Kathiriya I.S., Barnes R., Schluterman M.K., King I.N., Butler C.A., Rothrock C.R., Eapen R.S., Hirayama-Yamada K., Joo K. (2003). GATA4 mutations cause human congenital heart defects and reveal an interaction with TBX5. Nature.

[46] Ahmed I., Anjum F. (2021). Atrioventricular Septal Defect. https://www.ncbi.nlm.nih.gov/books/NBK562194/.

[47] Crawford F.A., Stroud M.R. (2001). Surgical repair of complete atrioventricular septal defect. Ann. Thorac. Surg..

[48] Robinson S.W., Morris C.D., Goldmuntz E., Reller M.D., Jones M.A., Steiner R.D., Maslen C.L. (2003). Missense mutations in CRELD1 are associated with cardiac atrioventricular septal defects. Am. J. Hum. Genet..

[49] Digilio M.C., Pugnaloni F., De Luca A., Calcagni G., Baban A., Dentici M.L., Versacci P., Dallapiccola B., Tartaglia M., Marino B. (2019). Atrioventricular canal defect and genetic syndromes. The unifying role of sonic hedgehog. Clin. Genet..

[50] Su B.H., Lin H.Y., Chiu H.Y., Tsai M.L., Chen Y.T., Lu I.C. (2020). Therapeutic strategy of patent ductus arteriosus in extremely preterm infants. Pediatr. Neonatol..

[51] Mani A., Meraji S.M., Houshyar R., Radhakrishnan J., Mani A., Ahangar M., Rezaie T.M., Taghavinejad M.A., Broumand B., Zhao H. (2002). Finding genetic contributions to sporadic disease. a recessive locus at 12q24 commonly contributes to patent ductus arteriosus. Proc. Natl. Acad. Sci. USA.

[52] Khetyar M., Syrris P., Tinworth L., Abushaban L., Carter N. (2008). Novel TFAP2B mutation in nonsyndromic patent ductus arteriosus. Genet. Test..

[53] Satoda M., Zhao F., Diaz G.A., Burn J., Goodship J., Davidson H.R., Pierpont M.E., Gelb B.D. (2000). Mutations in TFAP2B cause Char syndrome, a familial form of patent ductus arteriosus. Nat. Genet..

[54] Hajj H., Dagle J.M. (2012). Genetics of patent ductus arteriosus susceptibility and treatment. Semin. Perinatol..

[55] Bhansali S., Phoon C. (2021). Truncus Arteriosus. https://www.ncbi.nlm.nih.gov/books/NBK534774/.

[56] Naimo P.S., Konstantinov I.E. (2021). Surgery for truncus arteriosus: Contemporary practice. Ann. Thorac. Surg..

[57] McElhinney D.B., Driscoll D.A., Emanuel B.S., Goldmuntz E. (2003). Chromosome 22q11 deletion in patients with truncus arteriosus. Pediatr. Cardiol..

[58] Heathcote K., Braybrook C., Abushaban L., Guy M., Khetyar M.E., Patton M.A., Carter N.D., Scambler P.J., Syrris P. (2005). Common arterial trunk associated with a homeodomain mutation of NKX2.6. Hum. Mol. Genet..

[59] Warnes C.A. (2006). Transposition of the great arteries. Circulation.

[60] Turon-Vinas A., Riverola-de Veciana A., Moreno-Hernando J., Bartrons-Casas J., Prada-Martinez F.H., Mayol-Gomez J., Caffarena-Calvar J.M. (2014). Characteristics and outcomes of transposition of great arteries in the neonatal period. Rev. Esp. Cardiol..

[61] Megarbane A., Salem N., Stephan E., Ashoush R., Lenoir D., Delague V., Kassab R., Loiselet J., Bouvagnet P. (2000). X-linked transposition of the great arteries and incomplete penetrance among males with a nonsense mutation in ZIC3. Eur. J. Hum. Genet..

[62] Bamford R.N., Roessler E., Burdine R.D., Saplakoglu U., dela Cruz J., Splitt M., Goodship J.A., Towbin J., Bowers P., Ferrero G.B. (2000). Loss-of-function mutations in the EGF-CFC gene CFC1 are associated with human left-right laterality defects. Nat. Genet..

[63] Unolt M., Putotto C., Silvestri L.M., Marino D., Scarabotti A., Valerio M., Caiaro A., Versacci P., Marino B. (2013). Transposition of great arteries. new insights into the pathogenesis. Front. Pediatr..

[64] Adegbola A., Musante L., Callewaert B., Maciel P., Hu H., Isidor B., Picker-Minh S., Le Caignec C., Delle Chiaie B., Vanakker O. (2015). Redefining the MED13L syndrome. Eur. J. Hum. Genet..

[65] De Ita M., Cisneros B., Rosas-Vargas H. (2020). Genetics of transposition of great arteries: Between laterality abnormality and outflow tract defect. J. Cardiovasc. Transl. Res..

[66] Aboulhosn J., Child J.S. (2006). Left ventricular outflow obstruction. subaortic stenosis, bicuspid aortic valve, supravalvar aortic stenosis, and coarctation of the aorta. Circulation.

[67] Rodes-Cabau J., Miro J., Dancea A., Ibrahim R., Piette E., Lapierre C., Jutras L., Perron J., Tchervenkow C.I., Poirier N. (2007). Comparison of surgical and transcatheter treatment for native coarctation of the aorta in patients > or =1 year old. The Quebec native coarctation of the aorta study. Am. Heart J..

[68] Freylikhman O., Tatarinova T., Smolina N., Zhuk S., Klyushina A., Kiselev A., Moiseeva O., Sjoberg G., Malashicheva A., Kostareva A. (2014). Variants in the NOTCH1 gene in patients with aortic coarctation. Congenit. Heart Dis..

[69] Gravholt C.H., Backeljauw P. (2017). New international Turner syndrome guideline. a multi-society feat. Eur. J. Endocrinol..

[70] Sarkozy A., Conti E., Esposito G., Pizzuti A., Dallapiccola B., Mingarelli R., Marino B., Digilio M.C., Paoletti V. (2003). Nonsyndromic pulmonary valve stenosis and the PTPN11 gene. Am. J. Med Genet. Part A.

[71] Cuypers J.A., Witsenburg M., van der Linde D., Roos-Hesselink J.W. (2013). Pulmonary stenosis. update on diagnosis and therapeutic options. Heart.

[72] Williams D.S. (2006). Bicuspid aortic valve. J. Insur. Med..

[73] Mordi I., Tzemos N. (2012). Bicuspid aortic valve disease: A comprehensive review. Cardiol. Res. Pract..

[74] Otto C.M., Nishimura R.A., Bonow R.O., Carabello B.A., Erwin J.P., Gentile F., Jneid H., Krieger E.V., Mack M., McLeod C. (2021). 2020 ACC/AHA guideline for the management of patients with valvular heart disease. A Report of the American College of Cardiology/American Heart Association Joint Committee on clinical practice guidelines. J. Am. Coll. Cardiol..

[75] Garg V., Muth A.N., Ransom J.F., Schluterman M.K., Barnes R., King I.N., Grossfeld P.D., Srivastava D. (2005). Mutations in NOTCH1 cause aortic valve disease. Nature.

[76] Bravo-Jaimes K., Prakash S.K. (2020). Genetics in bicuspid aortic valve disease. Where are we?. Prog. Cardiovasc. Dis..

[77] Manning W.J. (2013). Asymptomatic aortic stenosis in the elderly. a clinical review. JAMA.

[78] Sugiyama K., Horigome H., Lin L., Murakami T., Shiono J., Yamashiro Y., Matsuura H., Yoda H., Yanagisawa H. (2019). Novel ELN mutation in a Japanese family with a severe form of supravalvular aortic stenosis. Mol. Genet. Genom. Med..

[79] Curran M.E., Atkinson D.L., Ewart A.K., Morris C.A., Leppert M.F., Keating M.T. (1993). The elastin gene is disrupted by a translocation associated with supravalvular aortic stenosis. Cell.

[80] Apitz C., Webb G.D., Redington A.N. (2009). Tetralogy of fallot. Lancet.

[81] Morgenthau A., Frishman W.H. (2018). Genetic origins of tetralogy of fallot. Cardiol. Rev..

[82] Silversides C.K., Lionel A.C., Costain G., Merico D., Migita O., Liu B., Yuen T., Rickaby J., Thiruvahindrapuram B., Marshall C.R. (2012). Rare copy number variations in adults with tetralogy of Fallot implicate novel risk gene pathways. PLoS Genet..

[83] Page D.J., Miossec M.J., Williams S.G., Monaghan R.M., Fotiou E., Cordell H.J., Sutcliffe L., Topf A., Bourgey M., Bourque G. (2019). Whole exome sequencing reveals the major genetic contributors to nonsyndromic tetralogy of fallot. Circ. Res..

[84] Javed R., Cetta F., Said S.M., Olson T.M., O’Leary P.W., Qureshi M.Y. (2019). Hypoplastic left heart syndrome: An overview for primary care providers. Pediatr. Rev..

[85] Saraf A., Book W.M., Nelson T.J., Xu C. (2019). Hypoplastic left heart syndrome. From bedside to bench and back. J. Mol. Cell. Cardiol..

[86] Dasgupta C., Martinez A.M., Zuppan C.W., Shah M.M., Bailey L.L., Fletcher W.H. (2001). Identification of connexin43 (alpha1) gap junction gene mutations in patients with hypoplastic left heart syndrome by denaturing gradient gel electrophoresis (DGGE). Mutat. Res..

[87] Yagi H., Liu X., Gabriel G.C., Wu Y., Peterson K., Murray S.A., Aronow B.J., Martin L.J., Benson D.W., Lo C.W. (2018). The Genetic landscape of hypoplastic left heart syndrome. Pediatr. Cardiol..

[88] Marin-Garcia J. (2009). Advances in molecular genetics of congenital heart disease. Rev. Esp. Cardiol..

[89] Lejeune J., Gautier M., Turpin R. (1959). Study of somatic chromosomes from 9 mongoloid children. C. R. Hebd. Seances. Acad. Sci..

[90] Lejeune J., Turpin R., Gautier M. (1959). Mongolism; a chromosomal disease (trisomy). Bull. Acad. Natl. Med..

[91] Kylat R.I. (2019). Tracheal stenosis and congenital heart disease in trisomy 21. Children.

[92] Zahari N., Mat Bah M.N., Hasliza A.R., Thong M.K. (2019). Ten-year trend in prevalence and outcome of Down syndrome with congenital heart disease in a middle-income country. Eur. J. Pediatr..

[93] Edwards J.H., Harnden D.G., Cameron A.H., Crosse V.M., Wolff O.H. (1960). A new trisomic syndrome. Lancet.

[94] Springett A., Wellesley D., Greenlees R., Loane M., Addor M.C., Arriola L., Bergman J., Cavero-Carbonell C., Csaky-Szunyogh M., Draper E.S. (2015). Congenital anomalies associated with trisomy 18 or trisomy 13. A registry-based study in 16 European countries, 2000-2011. Am. J. Med. Genet. Part A.

[95] Cereda A., Carey J.C. (2012). The trisomy 18 syndrome. Orphanet J. Rare. Dis..

[96] Patau K. (1960). The identification of individual chromosomes, especially in man. Am. J. Hum. Genet.

[97] Patau K., Smith D.W., Therman E., Inhorn S.L., Wagner H.P. (1960). Multiple congenital anomaly caused by an extra autosome. Lancet.

[98] Meyer R.E., Liu G., Gilboa S.M., Ethen M.K., Aylsworth A.S., Powell C.M., Flood T.J., Mai C.T., Wang Y., Canfield M.A. (2016). Survival of children with trisomy 13 and trisomy 18. A multi-state population-based study. Am. J. Med. Genet. Part A.

[99] Gravholt C.H., Viuff M.H., Brun S., Stochholm K., Andersen N.H. (2019). Turner syndrome: Mechanisms and management. Nat. Rev. Endocrinol..

[100] Morales-Demori R. (2017). Congenital heart disease and cardiac procedural outcomes in patients with trisomy 21 and Turner syndrome. Congenit. Heart Dis..

[101] Turner H. (1938). A Syndrome of infantilism, congenital webbed neck, and cubitus valgus. Endocrinology.

[102] Ford C.E., Jones K.W., Miller O.J., Mittwoch U., Penrose L.S., Ridler M., Shapiro A. (1959). The chromosomes in a patient showing both mongolism and the Klinefelter syndrome. Lancet.

[103] Ford C.E., Jones K.W., Polani P.E., De Almeida J.C., Briggs J.H. (1959). A sex-chromosome anomaly in a case of gonadal dysgenesis (Turner’s syndrome). Lancet.

[104] Ford C.E., Polani P.E., Briggs J.H., Bishop P.M. (1959). A presumptive human XXY/XX mosaic. Nature.

[105] Saliba A., Figueiredo A.C.V., Baroneza J.E., Afiune J.Y., Pic-Taylor A., Oliveira S.F., Mazzeu J.F. (2020). Genetic and genomics in congenital heart disease: A clinical review. J. Pediatr..

[106] DiGeorge A.M. (1968). Congenital Absence of the Thymus and Its Immunologic Consequences. Concurrence with Congenital Hypoparathyroidism.

[107] De la Chapelle A., Herva R., Koivisto M., Aula P. (1981). A deletion in chromosome 22 can cause DiGeorge syndrome. Hum. Genet..

[108] Andersen S.L., Laurberg P. (2014). Antithyroid drugs and congenital heart defects. ventricular septal defect is part of the methimazole/carbimazole embryopathy. Eur. J. Endocrinol..

[109] Edelmann L., Pandita R.K., Spiteri E., Funke B., Goldberg R., Palanisamy N., Chaganti R.S., Magenis E., Shprintzen R.J., Morrow B.E. (1999). A common molecular basis for rearrangement disorders on chromosome 22q11. Hum. Mol. Genet..

[110] Digilio M.C., Marino B. (2016). What is new in genetics of congenital heart defects?. Front. Pediatr..

[111] Mefford H.C., Sharp A.J., Baker C., Itsara A., Jiang Z., Buysse K., Huang S., Maloney V.K., Crolla J.A., Baralle D. (2008). Recurrent rearrangements of chromosome 1q21.1 and variable pediatric phenotypes. N. Engl. J. Med..

[112] Digilio M.C., Bernardini L., Consoli F., Lepri F.R., Giuffrida M.G., Baban A., Surace C., Ferese R., Angioni A., Novelli A. (2013). Congenital heart defects in recurrent reciprocal 1q21.1 deletion and duplication syndromes: Rare association with pulmonary valve stenosis. Eur. J. Med. Genet..

[113] Shapira S.K., McCaskill C., Northrup H., Spikes A.S., Elder F.F., Sutton V.R., Korenberg J.R., Greenberg F., Shaffer L.G. (1997). Chromosome 1p36 deletions. the clinical phenotype and molecular characterization of a common newly delineated syndrome. Am. J. Hum. Genet..

[114] Arndt A.K., Schafer S., Drenckhahn J.D., Sabeh M.K., Plovie E.R., Caliebe A., Klopocki E., Musso G., Werdich A.A., Kalwa H. (2013). Fine mapping of the 1p36 deletion syndrome identifies mutation of PRDM16 as a cause of cardiomyopathy. Am. J. Hum. Genet..

[115] Lubs H.A., Lubs L.M., Casperson T. (1973). New cytogenetic technics applied to a series of children with mental retardation. Nobel Symposium 23. Chromosome Identification Technique and Applications in Biology and Medicine.

[116] Wat M.J., Shchelochkov O.A., Holder A.M., Breman A.M., Dagli A., Bacino C., Scaglia F., Zori R.T., Cheung S.W., Scott D.A. (2009). Chromosome 8p23.1 deletions as a cause of complex congenital heart defects and diaphragmatic hernia. Am. J. Med. Genet. Part A.

[117] Andersen T.A., de Troelsen K.L., Larsen L.A. (2014). Of mice and men. molecular genetics of congenital heart disease. Cell. Mol. Life Sci..

[118] Kleefstra T., Brunner H.G., Amiel J., Oudakker A.R., Nillesen W.M., Magee A., Genevieve D., Cormier-Daire V., van Esch H., Fryns J.P. (2006). Loss-of-function mutations in euchromatin histone methyl transferase 1 (EHMT1) cause the 9q34 subtelomeric deletion syndrome. Am. J. Hum. Genet.

[119] Yatsenko S.A., Brundage E.K., Roney E.K., Cheung S.W., Chinault A.C., Lupski J.R. (2009). Molecular mechanisms for subtelomeric rearrangements associated with the 9q34.3 microdeletion syndrome. Hum. Mol. Genet..

[120] Wolf U., Reinwein H., Porsch R., Schroter R., Baitsch H. (1965). Deficiency on the short arms of a chromosome No. 4. Humangenetik.

[121] Hirschhorn K., Cooper H.L., Firschein I.L. (1965). Deletion of short arms of chromosome 4-5 in a child with defects of midline fusion. Humangenetik.

[122] Von Elten K., Sawyer T., Lentz-Kapua S., Kanis A., Studer M. (2013). A case of Wolf-Hirschhorn syndrome and hypoplastic left heart syndrome. Pediatr. Cardiol..

[123] Williams J.C., Barratt-Boyes B.G., Lowe J.B. (1961). Supravalvular aortic stenosis. Circulation.

[124] Beuren A.J., Apitz J., Harmjanz D. (1962). Supravalvular aortic stenosis in association with mental retardation and a certain facial appearance. Circulation.

[125] Del Pasqua A., Rinelli G., Toscano A., Iacobelli R., Digilio C., Marino B., Saffirio C., Mondillo S., Pasquini L., Sanders S.P. (2009). New findings concerning cardiovascular manifestations emerging from long-term follow-up of 150 patients with the Williams-Beuren-Beuren syndrome. Cardiol. Young.

[126] Kitagawa H., Fujiki R., Yoshimura K., Mezaki Y., Uematsu Y., Matsui D., Ogawa S., Unno K., Okubo M., Tokita A. (2003). The chromatin-remodeling complex WINAC targets a nuclear receptor to promoters and is impaired in Williams syndrome. Cell.

[127] Noonan J.A. (1968). Hypertelorism with Turner phenotype: A new syndrome with associated congenital heart disease. Am. J. Dis. Child..

[128] Jhang W.K., Choi J.H., Lee B.H., Kim G.H., Yoo H.W. (2016). cardiac manifestations and associations with gene mutations in patients diagnosed with RASopathies. Pediatr. Cardiol..

[129] Roberts A.E., Araki T., Swanson K.D., Montgomery K.T., Schiripo T.A., Joshi V.A., Li L., Yassin Y., Tamburino A.M., Neel B.G. (2007). Germline gain-of-function mutations in SOS1 cause Noonan syndrome. Nat. Genet..

[130] Pandit B., Sarkozy A., Pennacchio L.A., Carta C., Oishi K., Martinelli S., Pogna E.A., Schackwitz W., Ustaszewska A., Landstrom A. (2007). Gain-of-function RAF1 mutations cause Noonan and LEOPARD syndromes with hypertrophic cardiomyopathy. Nat. Genet..

[131] Costello J.M. (1977). A new syndrome. mental subnormality and nasal papillomata. Aust. Paediatr. J..

[132] Gripp K.W., Morse L.A., Axelrad M., Chatfield K.C., Chidekel A., Dobyns W., Doyle D., Kerr B., Lin A.E., Schwartz D.D. (2019). Costello syndrome. Clinical phenotype, genotype, and management guidelines. Am. J. Med. Genet. Part A.

[133] Gripp K.W., Rauen K.A., Adam M.P., Ardinger H.H., Pagon R.A., Wallace S.E., Bean L.J.H., Mirzaa G., Amemiya A. (1993). Costello syndrome. GeneReviews^®^.

[134] Tidyman W.E., Rauen K.A. (2009). The RASopathies. developmental syndromes of Ras/MAPK pathway dysregulation. Curr. Opin. Genet. Dev..

[135] Gorlin R.J., Anderson R.C., Blaw M. (1969). Multiple lentigenes syndrome. Am. J. Dis. Child..

[136] Gelb B.D., Roberts A.E., Tartaglia M. (2015). Cardiomyopathies in Noonan syndrome and the other RASopathies. Prog. Pediatr. Cardiol..

[137] Holt M., Oram S. (1960). Familial heart disease with skeletal malformations. Br. Heart J..

[138] Geng J., Picker J., Zheng Z., Zhang X., Wang J., Hisama F., Brown D.W., Mullen M.P., Harris D., Stoler J. (2014). Chromosome microarray testing for patients with congenital heart defects reveals novel disease causing loci and high diagnostic yield. BMC Genom..

[139] Patel C., Silcock L., McMullan D., Brueton L., Cox H. (2012). TBX5 intragenic duplication. a family with an atypical Holt-Oram syndrome phenotype. Eur. J. Hum. Genet..

[140] Adams F.H., Oliver C.P. (1945). Hereditary deformities in man due to arrested development. J. Hered..

[141] Digilio M.C., Marino B., Baban A., Dallapiccola B. (2015). Cardiovascular malformations in Adams-Oliver syndrome. Am. J. Med. Genet. Part A.

[142] Watson G.H., Miller V. (1973). Arteriohepatic dysplasia. familial pulmonary arterial stenosis with neonatal liver disease. Arch. Dis. Child..

[143] Alagille D., Odievre M., Gautier M., Dommergues J.P. (1975). Hepatic ductular hypoplasia associated with characteristic facies, vertebral malformations, retarded physical, mental, and sexual development, and cardiac murmur. J. Pediatr..

[144] Zhang E., Xu Y., Yu Y., Chen S., Yu Y., Sun K. (2018). JAG1 lossoffunction mutations contributed to Alagille syndrome in two Chinese families. Mol. Med. Rep..

[145] Niikawa N., Matsuura N., Fukushima Y., Ohsawa T., Kajii T. (1981). Kabuki make-up syndrome. a syndrome of mental retardation, unusual facies, large and protruding ears, and postnatal growth deficiency. J. Pediatr..

[146] Kawame H., Hannibal M.C., Hudgins L., Pagon R.A. (1999). Phenotypic spectrum and management issues in Kabuki syndrome. J. Pediatr..

[147] Pagon R.A., Downing A.L., Ruvalcaba R.H. (1986). Kabuki make-up syndrome in a Caucasian. Ophthalmic Paediatr. Genet..

[148] Ng S.B., Bigham A.W., Buckingham K.J., Hannibal M.C., McMillin M.J., Gildersleeve H.I., Beck A.E., Tabor H.K., Cooper G.M., Mefford H.C. (2010). Exome sequencing identifies MLL2 mutations as a cause of Kabuki syndrome. Nat. Genet..

[149] Paulussen A.D., Stegmann A.P., Blok M.J., Tserpelis D., Posma-Velter C., Detisch Y., Smeets E.E., Wagemans A., Schrander J.J., van den Boogaard M.J. (2011). MLL2 mutation spectrum in 45 patients with Kabuki syndrome. Hum. Mutat..

[150] Hall B.D. (1979). Choanal atresia and associated multiple anomalies. J. Pediatr..

[151] Hittner H.M., Hirsch N.J., Kreh G.M., Rudolph A.J. (1979). Colobomatous microphthalmia, heart disease, hearing loss, and mental retardation—A syndrome. J. Pediatr. Ophthalmol. Strabismus.

[152] Jongmans M.C., Admiraal R.J., van der Donk K.P., Vissers L.E., Baas A.F., Kapusta L., van Hagen J.M., Donnai D., de Ravel T.J., Veltman J.A. (2006). CHARGE syndrome. the phenotypic spectrum of mutations in the CHD7 gene. J. Med. Genet..

[153] Leon L.E., Benavides F., Espinoza K., Vial C., Alvarez P., Palomares M., Lay-Son G., Miranda M., Repetto G.M. (2017). Partial microduplication in the histone acetyltransferase complex member KANSL1 is associated with congenital heart defects in 22q11.2 microdeletion syndrome patients. Sci. Rep..

[154] Koolen D.A., Morgan A., de Vries B.B.A., Adam M.P., Ardinger H.H., Pagon R.A., Wallace S.E., Bean L.J.H., Mirzaa G., Amemiya A. (1993). Koolen-de Vries Syndrome. GeneReviews^®^.

[155] Koolen D.A., Pfundt R., Linda K., Beunders G., Veenstra-Knol H.E., Conta J.H., Fortuna A.M., Gillessen-Kaesbach G., Dugan S., Halbach S. (2016). The Koolen-de Vries syndrome. a phenotypic comparison of patients with a 17q21.31 microdeletion versus a KANSL1 sequence variant. Eur. J. Hum. Genet..

[156] Jacobsen P., Hauge M., Henningsen K., Hobolth N., Mikkelsen M., Philip J. (1973). An (11;21) translocation in four generations with chromosome 11 abnormalities in the offspring. A clinical, cytogenetical, and gene marker study. Hum. Hered..

[157] Tassano E., Janis S., Canepa A., Zanotto E., Torello C., Gimelli G., Cuoco C. (2016). Interstitial 11q24 deletion. a new case and review of the literature. J. Appl. Genet..

[158] Davidson H.R. (1993). A large family with patent ductus arteriosus and unusual face. J. Med. Genet..

[159] Gelb B.D., Adam M.P., Ardinger H.H., Pagon R.A., Wallace S.E., Bean L.J.H., Mirzaa G., Amemiya A. (1993). Char syndrome. GeneReviews^®^.

[160] Myhre S.A., Ruvalcaba R.H., Graham C.B. (1981). A new growth deficiency syndrome. Clin. Genet..

[161] Starr L.J., Lindor N.M., Lin A.E., Adam M.P., Ardinger H.H., Pagon R.A., Wallace S.E., Bean L.J.H., Mirzaa G., Amemiya A. (1993). Myhre Syndrome. GeneReviews^®^.

[162] Ruiz-Perez V.L., Ide S.E., Strom T.M., Lorenz B., Wilson D., Woods K., King L., Francomano C., Freisinger P., Spranger S. (2000). Mutations in a new gene in Ellis-van Creveld syndrome and Weyers acrodental dysostosis. Nat. Genet..

[163] McKusick V.A., Egeland J.A., Eldridge R., Krusen D.E. (1964). Dwarfism in the Amish I: The Ellis-Van Creveld Syndrome. Bull. Johns Hopkins Hosp..

[164] Baujat G., Le Merrer M. (2007). Ellis-van Creveld Syndrome. Orphanet J. Rare Dis..

